# The Overall Quality of Life and Oncological Outcomes Following Radical Hysterectomy in Cervical Cancer Survivors Results from a Large Long-Term Single-Institution Study

**DOI:** 10.3390/cancers14020317

**Published:** 2022-01-09

**Authors:** Mihai Stanca, Dan Mihai Căpîlna, Cristian Trâmbițaș, Mihai Emil Căpîlna

**Affiliations:** 1 First Obstetrics and Gynecology Clinic, University of Medicine, Pharmacy, Science and Technology “G.E. Palade” of Târgu Mureș, Gheorghe Marinescu Street, Number 38, 540142 Targu Mures, Romania; capilnadan@gmail.com (D.M.C.); mcapilna@gmail.com (M.E.C.); 2 Anatomy and Embryology Department, University of Medicine, Pharmacy, Science and Technology “G.E. Palade” of Târgu Mureș, Gheorghe Marinescu Street, Number 38, 540142 Targu Mures, Romania; cristian.trambitas@umfst.ro

**Keywords:** long-term cervical cancer survival, disease-free survivors, quality of life, QLQ-C30, QLQ-CX24, sexual functioning, prognostic factors

## Abstract

**Simple Summary:**

Romania maintains its regrettably leading position in terms of mortality caused by cervical cancer in Europe, with any available studies evaluating the oncological outcomes and quality of life of these patients. Our study could provide a historical comparison for future randomized controlled trials in Eastern Europe needed to confirm these results.

**Abstract:**

(1) Background: Cervical cancer patients have been found to have worse quality of life (QoL) scores due to cancer treatment, not only when compared to the general population, but also when compared to other gynecological cancer survivors. In Eastern European developing countries, the health care system often cannot afford the uppermost standardized treatment for these patients. In the absence of a comparable study in our country, the authors’ aim for this retrospective cross-sectional observational study was to evaluate the overall survival (OS) and the QoL o cervical cancer survivors; (2) Methods: 430 patients were analyzed. The first objective is to evaluate the OS rates of patients with cervical cancer stages IA2 to IIB undergoing radical hysterectomy (RH) +/− neoadjuvant or adjuvant radiotherapy +/− chemoradiotherapy treatment combinations. The second objective is to assess their QoL, using two standardized questionnaires issued by the European Organisation for Research and Treatment of Cancer (EORTC), namely QLQ-C30 and QLQ-CX24. (3) Results: The mean age of the participants was 51 years (22–76) and the average follow-up time was 65 months (2–128). At the time of the analysis, 308 out of 430 patients were alive, with a mean five-year OS of 72.4%. The multivariate Cox regression analysis identified stage IIB, parametrial invasion, and the lymph node metastases as independent prognostic risk factors negatively impacting the OS. Of the 308 patients still alive at the time of the analysis, 208 (68%) answered the QoL questionnaires. The QLQ-C30 shows a good long-term Global QoL of 64.6 (median), good functioning scores, and a decent symptom scale value. However, the EORTC QLQ-CX24 showed high values of cervical cancer-specific symptoms, namely: lymphedema, peripheral neuropathy, severe menopausal symptoms, and distorted body-image perception. The results also indicate a significant decline in the quality of sexual life with a low sexual enjoyment and decreased level of sexual activities. (4) Conclusion: Despite a good OS, in this setting of patients, cervical cancer survivors have a modest QoL and sexual function. Our study may provide a comparison for future randomized, controlled trials in Eastern European countries needing to confirm these results.

## 1. Introduction

Cervical cancer is the second most common gynecologic cancer, with more than a quarter of cases diagnosed as locally advanced disease, and with a five-year survival rate of 60% [1].

Many cervical cancer patients are young, socially and sexually active women whose quality of life (QoL) is at risk of being endangered by treatment, whether it is surgery, radiotherapy (RT), chemotherapy (CT), or a combination of these. The survivors might spend the rest of their lives with the side effects of cancer treatment, thereby decreasing their QoL [2,3].

Women with early-stage cervical cancer are primarily treated with radical hysterectomy (RH) +/− adjuvant RT/CT or adjuvant concurrent chemoradiotherapy (CCRT), depending on the histopathological assessment [4]. In locally advanced cervical cancer (LACC), definitive chemoradiotherapy (CRT) is suggested, whereas the role of neoadjuvant chemoradiotherapy CRT + RH remains controversial [5,6,7]. Despite superior survival rates and disease control, the safety of the multimodal therapies is ambiguous and may be linked to severe toxicity, lessening the QoL [8]. Therefore, treatment planning should not strictly aim to increase the Overall Survival (OS), but should also focus on QoL.

The annual incidence of cervical cancer in Eastern Europe is 36,000 cases, while in Western and Southern Europe it is around 9000 cases and less than 6000 registered per year in Northern Europe [9]. Although efforts are being made to alleviate this situation, Romania maintains its regrettably leading position in terms of mortality caused by this disease [10].

Due to this worrying situation in Eastern Europe and the absence of a comparable study in our country, the authors aim for two objectives. The first evaluates the OS rates of patients with clinical cervical cancer stages IA2 to IIB (FIGO 2018 [11]) undergoing type C2/Piver III RH +/− neoadjuvant or adjuvant RT +/− CT treatment combinations, depending on demographic, clinical, surgical, therapeutic, and histopathological features. The second objective is to assess their QoL using two translated standardized questionnaires issued by the European Organisation for Research and Treatment of Cancer (EORTC), namely QLQ-C30 and QLQ-CX24.

## 2. Materials and Methods

The study was conducted with the authorization of the Ethical Committee (approval code: 34535) and written informed consent was attained from all patients.

We obtained the consent of the EORTC to use the QLQ-C30 and QLQ-CX24 questionnaires for the present study.

### 2.1. Patient Population and Study Design

In this study, compared to the previous one conducted by the authors [12], the research range has been expanded with another 208 patients to perform a more exhaustive statistical analysis, allowing for a broader horizon concerning the impact of cervical cancer on OS and QoL.

In the current retrospective cross-sectional observational study, the authors included 430 patients who had undergone type C2/Piver III RH +/− neoadjuvant or adjuvant RT +/− CT regimens for cervical cancer stages IA2-IIB (FIGO 2018). All surgeries were performed by a team of highly trained gynecologic oncologists [13].

The demographic, surgical, histopathological, and oncological data were acquired from the patient’s files and will be presented in detail (Table 1).

The study has two main objectives. The first one is to assess the five-year OS, described as the period from surgical procedure to the last follow-up visit or death, and the impact of various treatment formulas and prognostic factors on survival. The second objective consists in the evaluation of the QoL of the surviving patients, assessed using the questionnaires EORTC QLQ-C30 and EORTC QLQ-CX24 [14,15].

After accomplishing the treatments, the patients had regular follow-up visits at an interval of three months in the first two years, every six months until five years, and then annually until the fall of 2020. A Pap smear and pelvic ultrasound examination were performed at each medical check-up appointment, as were annual chest and abdominal Computed Tomography or Magnetic Resonance Imaging tests. The deaths caused by cervical cancer were documented by phone or through postal cards obtained from the patients’ relatives.

### 2.2. Inclusion and Exclusion Criteria

The inclusion criteria consisted of the following: age ≥ 18 years old, patients with histologically and clinically proven cervical cancer FIGO stage IA2-IIB who mandatorily underwent RH +/− neoadjuvant or adjuvant RT +/− CT regimens, with a follow-up extent of at least 24 months. Deceased patients or patients with recurrent disease or other malignancies and known pathologies were excluded from the QoL study altogether with the patients who expressed their refusal to participate or lost to follow-up.

### 2.3. Treatments

Patients included in the study underwent the following treatment options: RH alone; neoadjuvant CRT followed by RH; neoadjuvant RT followed by RH; adjuvant CCRT following RH [12], and adjuvant CT following RH. The type of surgery was C2/Piver III RH with pelvic lymph node dissection. Concisely, regarding the RT regimen, the RT beam consisted of a fraction of 1.8 Gy tumor dose every day with five fractions weekly with an average intake dosage of 58.5 Gy. In terms of CT, patients received weekly Cisplatin +/− 5-fluorouracil during RT.

### 2.4. Statistical Analysis and Data Assessment

Variances among categorical variables were established using the χ^2^ test. Differences among variables were analyzed by *t*-test. *p* < 0.05 was considered statistically significant.

The OS was assessed in correspondence with the Kaplan-Meier method. The survival length was defined from the day of surgery to the last contact. The endpoint for the OS was death. The survival was compared via the log-rank test, and risk factors were evaluated using the Cox regression examination. A proportional hazards model was operated to carry out a multivariable Cox analysis of various factors that impacts the OS with 95% confidence intervals (95% CI). We used IBM SPSS software 23.0 for the data analysis.

### 2.5. Questionnaires

All alive patients who met the inclusion criteria received a postal letter containing the informed consent and a set of EORTC validated self-administered questionnaires, all together with a reply-paid envelope. Patients who did not respond within three months were resent the same postal letter and called one month later.

The EORTC QLQ-C30 questionnaire includes 30 items. This is a basic instrument universally used for all types of malignancies which consists of five functional scales (physical, role, emotional, cognitive, and social), three symptom scales (fatigue, pain, and nausea/vomiting), a global health QoL scale, and six single items assessing symptoms often declared by cancer patients (dyspnea, appetite loss, sleep disturbance, constipation, and diarrhea), and the supposed financial problem [14].

The EORTC QLQ-CX24 is a cervical cancer-specific explicit questionnaire, related to the previous one, which includes 24 items concerning symptoms that might result from cervical cancer therapy. It has four functioning scales: two multi-item scales (body image and sexual/vaginal functioning) and two single-item scales (sexual activity and sexual enjoyment), also five symptom scales, four single-item scales (lymphedema, peripheral neuropathy, menopausal symptoms, and sexual worry), and one multi-item scale (symptom experience) [15]. Both questionnaires use a 4-point answer scale as follows: “Not at all”, “A little”, “Quite a bit” and “Very much” to independently evaluate functional or symptom items, and a 7-point answer for each functional or symptom item, and also a 7-point answer, from poor to excellent [14,15].

To facilitate the interpretation, the scales and items of the questionnaires were converted to a 0 to 100 scale using a scoring manual [14]. For EORTC QLQ-C30, a higher score for the global QoL and functional scales suggested appropriate functioning or good QoL, whereas for the items and symptom scales a higher score relates to higher intensity disturbances [14]. For QLQ-CX24, higher scores indicate severe symptoms and worse functioning except for sexual activity and sexual satisfaction [15]. All statistical outcomes were described in raw numbers, rates, summarized as a mean (SD) (95% standard error), and medians [ranges].

## 3. Results

### 3.1. Patient Characteristics and Follow-Up

We identified 430 cervical cancer patients who met the eligibility criteria who underwent RH in the First Obstetrics and Gynecology Clinic of Târgu Mureș, Romania from January 2010 to March 2019.

The mean age of the participants was 51 years (range 22–76). Tumor descriptions revealed FIGO 2018 clinical stages [11]: IA1 0.7%, IB1 2.8%, IB2 32.6%, IB3 14%, IIA1 8.6%, IIA2 6.5%, IIB 34.9%; squamous cell carcinoma was encountered in 83.3% of cases, followed by adenocarcinoma with 10% and adenosquamous carcinoma with 5.3%. Regarding negative prognostic factors, 122 patients (28.4%) had parametrial invasion, 79 patients (18.4%) had positive resection margins, and 138 patients (32.1%) had lymph node metastases.

Concerning treatment regimens, 57 patients (13.3%) underwent surgery only, 46 patients (10.7%) received neoadjuvant RT followed by surgery, 86 patients (20%) received neoadjuvant CRT followed by surgery, 222 patients (34%) were given adjuvant CCRT following surgery [12] and 19 patients (4.4%) received adjuvant CT following surgery. All the information is detailed in Table 1.

### 3.2. Survey Results

A thorough analysis of the 222 patients who underwent RH followed by adjuvant CCRT is described in the prior published study [12].

Patient follow-up lasted until 1 November 2020, with an average follow-up of 65 months (2–128). At the time of the analysis, 308 out of 430 patients were alive, with a mean five-year OS of 72.4% (Table 1). The major intraoperative complications were avoided [16]. No death caused by surgery or RT/CT was registered. Among the survivors, 22 had recurrent disease, hence were excluded from the QoL analysis.

**Figure 1:**
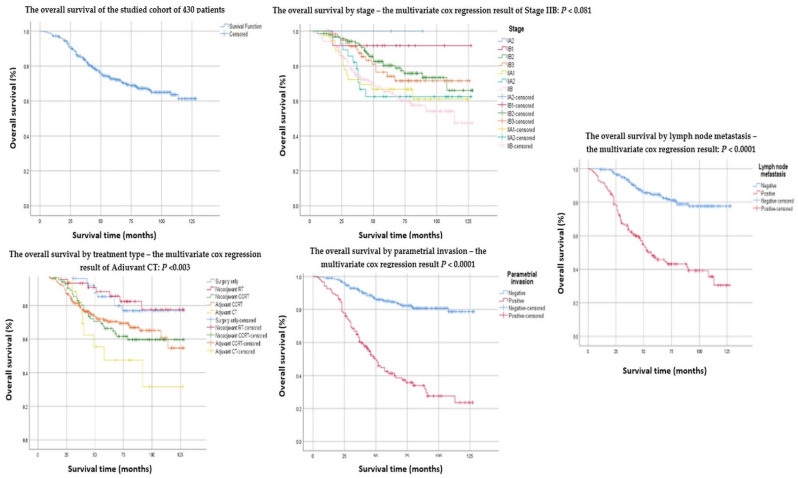
The Kaplan Mayer survival curves.

The univariate analysis shows that OS was influenced by FIGO clinical stages IB1, IIB, and the outer 1/3 cervical stromal invasion. Also, patients who received only surgery, neoadjuvant RT followed by surgery, or adjuvant CT following surgery had a negative prognostic, with *p* < 0.05 (Table 1) (Figure 1). Although the univariate analysis identifies several negative prognostic factors (Table 1), the multivariate Cox examination only recognizes stage IIB, lymph node metastases, and parametrial invasion as self-determining prognostic factors negatively impacting the OS. Additionally, adjuvant CT following surgery is associated with poor survival compared to other treatment regimens used (Table 2).

Regarding the disease recurrences, the clinical-stage FIGO IIB is an independent negative prognostic factor with *p* < 0.02 [12] (Table 1).

### 3.3. QoL Results

Out of the 308 alive patients, 68% (*n* = 208) answered the QoL self-assessment questionnaires. In most cases, the failure to contact the patients was due to address or phone number changes, or we came across the refusal to participate and to supply personal information (data not shown). As mentioned, the 22 patients with recurrent disease were excluded for QoL assessment.

Therefore, the QoL study population included 208 women with a mean age of 52 years (range 22–60). Of these, 123 patients (59%) received adjuvant CCRT, 29 patients (14%) received neoadjuvant CRT, 29 patients (14%) received RH only, 21 patients (10%) received neoadjuvant RT, and only six patients (3%) received adjuvant CT. The questionnaires were sent to the patients after an average follow-up of 48 months subsequent to the ending of the treatment.

**Figure 2:**
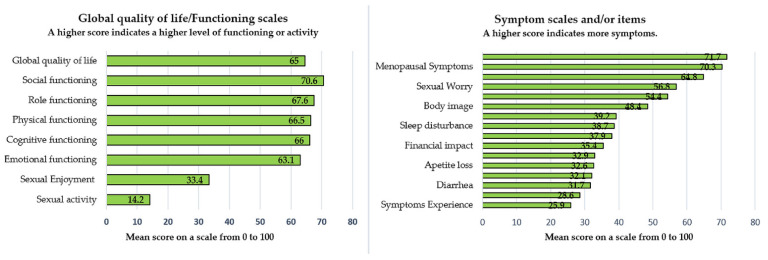
The QoL of the 208 patients who answered the EORTC QLQ-C30 and EORTC QLQ-CX24.

Regarding the EORTC QLQ-C30 [14] which is a basic questionnaire universally used for all types of malignancies, the survivors revealed a relatively good global QoL of 64.6 (median). This refers to the perception of wellbeing in the week they fulfilled the questionnaires. The functional status represented by physical, role, cognitive, emotional, and social functioning had also satisfactory scores of 66.5, 67.5, 66.2, 63.1, and 70.6, respectively, symbolizing good functioning and good QoL. The symptoms scales consisting of nausea/vomiting, dyspnea, pain, diarrhea, loss of appetite, and financial difficulty showed a relatively low level of side effects of cancer treatment. However, some of the symptoms that most frequently caused discomfort but rarely led to major difficulties were constipation, insomnia, and fatigue, with values corresponding to 39.2, 38.7, and 37.9 (Table 3) (Figure 2).

The EORTC QLQ-CX24 questionnaire measures the specific symptoms of cervical cancer. The symptoms experience showed a good result with a value of 25.9, although the body image, lymphedema, peripheral neuropathy, and menopausal symptoms had scores of 48.4 ± 31.3, 54.4 ± 31.6, 71.7 ± 30.4, 70.3 ± 32.3, and 56.8 ± 34.6, respectively, showing a high-level of cervical cancer-specific symptoms following oncological therapy (Table 3) (Figure 2).

Concerning sexual activity, data indicated an unsatisfying level of sexual enjoyment with a worsening of sexual activity with scores of 64.8 ± 23.7, 56.8 ± 34.6, 14.2 ± 25.6, and 33.4 ± 28.2, respectively, with regard to sexual/vaginal functioning, sexual worry, sexual activity, and sexual enjoyment.

## 4. Discussion

Globally, cervical cancer is the second most common type of cancer among female malignancies, with high mortality in developing countries [18]. Although the treatment of cervical cancer has been developed, there are still significant negative consequences for the patients [19]. The importance of measuring the QoL arises from the knowledge that cervical cancer patients have been found to have worse QoL scores due to cancer treatment, not only when compared to the general population, but also when compared with other gynecological cancer survivors [20].

Although definitive CRT is considered to be the therapy of choice for LACC, the role of surgery remains controversial [5,12,21]. As for neoadjuvant RT before surgery, it is recommended for tumor shrinking, allowing for better surgical resection [22,23], while neoadjuvant CT increases the sensitivity to radiation of the malignant cells [24]. Tongqing et al. [25] compared the outcomes of patients who were treated with neoadjuvant RT followed by surgery to those who received only surgery, achieving similar five-year OS between the two groups (80% vs. 74.7%). Benedetti-Panici et al. [26], showed that neoadjuvant CT and neoadjuvant CRT administered before surgery can achieve better OS compared to patients only undergoing surgery [26]. However, in the current study, the patients who had undergone surgery alone obtained a higher five-year OS of 79.9%, than the neoadjuvant RT + surgery group with 77.6% and the neoadjuvant CRT + surgery group with 66.4%. This result may have occurred since the patients with the early-stage disease were treated with surgery only, compared to the other two groups of patients with LACC who required neoadjuvant treatment before surgery, hence the inferior OS.

The surgery has to be followed by adjuvant therapy to increase local control when pathological risk factors are identified [27,28,29]. In the current study, the adjuvant CCRT following surgery achieved higher survival rates than patients treated with adjuvant CT with a five-year OS of 69.9% vs. 47.5%, the multivariate analysis linking the adjuvant CT with worse OS (*p* < 0.03).

Concerning the histopathological features that negatively influenced the OS, the univariate analysis identified several elements (Table 1), although the multivariate Cox regression analysis isolates only FIGO clinical-stage IIB, the parametrial invasion, and the lymph node metastases as independent risk factors, influencing survival (Table 2).

Functional elements of QoL such as social and psychological traits and those related to body image and sexual perception are commonly connected with various cervical cancer treatment regimens, with a negative impact on QoL [15]. Therefore, in 70% of patients, type C2/Piver III RH leads to hypogastric plexus injury with voiding dysfunction (mixed urinary incontinence, overactive detrusor) and nonetheless to colorectal, sexual and pelvic floor dysfunction [2,30,31,32,33,34]. RT can induce long-term toxicity, impairment in urinary, gastrointestinal, and sexual function with vaginal shortening and atrophy leading to dyspareunia and loss of sexual desire, while CT can induce diarrhea, constipation, nausea and vomiting, hormonal fluctuations, etc. [35,36,37]. To prevent the atrophic process that impacts sexual function, an early start of local estrogen after therapy might be of importance [38].

Concisely, there is no ultimate therapeutic choice that fully preserves the QoL of cervical cancer patients, as every single treatment more or less induces low self-esteem, variations in self-image, marital conflicts, and anxieties [39]. Corresponding to a larger study, sexual function is the main cause of symptom-induced concerns subsequent to any type of therapy for cervical cancer [31].

It appears that the QoL is not so much influenced by the stage of the disease as much as it is by the treatment used [40]. Patients receiving RH with RT +/− CT are subjected to twice as many side effects as those who underwent surgery only [17,31,32,34]. Some might state that patients subjected to laparoscopic surgery would get better results, but recently Frumovitz et al. [41] rejected this hypothesis by stating that it is irrelevant regarding QoL whether the RH is administered by open or minimally invasive surgery. Additionally, laparoscopic surgery has been linked with lower rates of disease-free survival and overall survival than open abdominal radical hysterectomy amongst women with early-stage cervical cancer [42].

This study aimed to assess the OS and the QoL of surviving patients using the validated questionnaires EORTC QLQ-C30 and EORTC QLQ-CX24. To our knowledge, this is the first study of its kind in Romania and other Eastern European countries.

In this study, 86% of patients were treated with RH and RT +/− CT and only 14% with RH alone. The research reports a good long-term Global QoL of 64.6 (median) and a five-year OS of 72.4%, which are good functioning scores and decent symptom scale values (Table 3) (Figure 2). These outcomes are consistent with other published studies [2,43,44,45,46,47].

However, the EORTC QLQ-CX24 cervical cancer-specific questionnaire showed high values of specific symptoms of this malignancy, namely lymphedema, peripheral neuropathy, severe menopausal symptoms, and a distorted body-image perception (Table 3) (Figure 2). The increased rate of lymphedema may be overcome by using sentinel lymph node biopsy [48]. The results also indicate a significant decline in the quality of sexual life with a low sexual enjoyment and a decreased level of sexual activities, scoring 64.8 ± 23.7, 56.8 ± 34.6, 14.2 ± 25.6, and 33.4 ± 28.2, respectively, for sexual/vaginal functioning, sexual worry, sexual activity, and sexual enjoyment. The results achieved are slightly worse, but consistent with other reports [2,36,37,43,44,45,46,47,49,50], proving the deteriorating of all functioning and symptom scales among patients undergoing RH with or without RT +/− CT, compared to control groups. Women and their partners ought to be helped in retrieving sexual healthcare services for effective treatment plans [51].

### Strength and Limitations

One of the strengths of this research is its uniqueness in Romania and other Eastern European countries. The study is divided into two main objectives, including a large number of patients enrolled (*n* = 430), followed for an average of 65 months (2–128), with all surgeries carried out at a single institution by the same gynecologic oncologists’ team. The first objective was to identify a wide range of outcomes that influenced the OS for which a thorough analysis of all demographic, clinical, surgical, histological, and oncological therapeutic records was conducted. The second objective was to assess the QoL of the survivors. 208 (68%) out of the 308 alive patients answered the EORTC QLQ-C30 and the EORTC QLX-CX24 validated questionnaires. Despite the heterogeneity of the studied group, the analyses accomplish to provision of a QoL depiction, which is slightly worse than the QoL of other studies conducted in Central, Western, and Northern Europe [2,36,37,43,44,45,46,47,49,50].

The limitations arise from its cross-sectional retrospective observational study nature. There is heterogeneity in terms of the treatment received. The study has no control group (e.g., healthy participants) or a pretreatment assessment of the QoL. Also, the authors failed to rule out associated pathologies of the patients. Furthermore, the QoL has not been separately analyzed corresponding to the treatment regimen received. Therefore, the results may not be extremely accurate, although they can be compared with other prospective control studies [2,36,37,43,44,45,46,47,49,50].

## 5. Conclusions

This study demonstrated a five-year OS of 72.4% in cervical cancer patients. The COX multivariate analysis identified FIGO clinical stage IIB, parametrial invasion, and lymph node metastases as determining factors for poor oncological outcomes. The QoL analysis indicated a good global QoL, with satisfactory functional, social, and symptoms score, though a high prevalence of cervical cancer-specific symptoms such as lymphedema, peripheral neuropathy, severe menopausal symptoms, body image distortion, reduced sexual activity, and lower sexual enjoyment were the most disabling treatment-related consequences.

Despite some limitations, this study demonstrated that properly treated patients achieved a good five-years OS, but with negative repercussions on QoL. Hence, it can be concluded that current cervical cancer survivors in our country have a modest-QoL and sexual function, raising a signal that these patients need healthcare counseling.

Regardless of national screening programs, Romania maintains its regrettably leading position in terms of mortality caused by cervical cancer [10]. The low screening compliance arises due to both misinformation and religious beliefs.

Our study could provide a historical comparison for future randomized controlled trials in Eastern Europe needed to confirm these results.

## Figures and Tables

**Table 1:** The demographic, clinical aspects, and the univariate overall survival analysis of the 430 patients included in the study.

	Number (%) or Median (Range)	Overall Survival				Recurrences	
		5-Year Survival Rate	95% CI	Mean Survival (Months)	*p* Value	Number	*p* Value
**No. of patients**	430				0.792	22	
**Age (years)**	51 (22–76)						
Under 30	3 (0.7%)	66.7%	69.4–63.9	58.6	0.657		
30–40	71 (16.5%)	70.8%	71.3–69.7	94.5	0.822		
41–50	121 (28.1%)	77.0%	77.4–76.5	101.8	0.474		
51–60	152 (35.3%)	73.3%	73.6–72.9	97.9	0.262		
61–70	79 (18.4%)	63.2%	63.8–62.5	89.8	0.355		
71–80	4 (0.9%)	50.0%	52.5–47.5	97.4	0.563		
**Provenance**					0.662		
Urban	180 (41.9%)	72.3%	72.6–71.9	99.0		12	
Rural	250 (58.1%)	72.6%	72.9–72.3	96.3		10	
**Clinical Stage (FIGO 2018)**							
IA2	3 (0.7%)	100%			0.559		
IB1	12 (2.8%)	91.1%	91.9%	117.9	0.171		
IB2	140 (32.6%)	80.3%	80.6–79.9	105.3	**0.011**	4	0.060
IB3	60 (14.0%)	74.2%	74.8–73.5	96.5	0.337	4	0.518
IIA1	37 (8.6%)	66.7%	67.4–65.9	89.7	0.463	2	0.615
IIA2	28 (6.5%)	62.5%	63.4–63.4	91.7	0.538	1	0.415
IIB	150 (34.9%)	64.5%	64.9–64.0	87.4	**0.001**	11	**0.020**
**Tumor size**					0.873		0.475
<4 cm	328 (76.3%)	73.0%	73.2–72.7	97.0		16	
≥4 cm	102 (23.7%)	70.8%	71.2–70.3	97.5		6	
**Histology**							
Squamous Cell Carcinoma	358 (83.3%)	73.0%	73.2–72.7	98.2	0.339	18	0.572
Adenocarcinoma	43 (10.0%)	70.1%	70.8–69.3	99.6	0.837	2	0.537
Adenosquamous	23 (5.3%)	67.2%	68.2–66.1	85.2	0.279		
Other	6 (1.4%)	50.0%	52.0–47.9	50.6	0.097	2	0.554
**Tumor differentiation grade**							
Grade 1 (well-differentiated)	67 (15.6%)	82.7%	83.1–82.2	105.1	0.074	1	0.615
Grade 2 (moderately-differentiated)	178 (41.4%)	70.7%	71.0–70.3	97.1	0.064	11	0.135
Grade 3 (poorly-differentiated)	185 (43.0%)	66.2%	66.5–65.8	94.3	0.848	9	0.141
**Depth of cervical stromal invasion**							
Inner 1/3	81 (18.8%)	84.3%	84.7–83.8	113.4	0.091	1	0.586
Middle 1/3	91 (21.2%)	80.9%	81.3–80.4	101.8	0.244	1	0.238
Outer 1/3	258 (60.0%)	65.8%	66.1–65.4	90.7	**0.001**	20	0.200
**Lymphovascular space invasion**					**0.0001**		0.207
Positive	263 (61.2%)	64.4%	64.7–64.0	87.7		19	
Negative	167 (38.8%)	85.4%	85.6–85.1	110.7		3	
**Parametrial involvement**					**0.0001**		0.463
Positive	122 (28.4%)	42.5%	42.9–42.0	64.4		10	
Negative	308 (71.6%)	85.1%	85.3–84.8	111.2		12	
**Resection margin status**					**0.0001**		0.109
Positive	79 (18.4%)	53.1%	53.7–52.4	80.5		8	
Negative	351 (81.6%)	77.2%	77.4–76.9	101.2		14	
**Pelvic lymph nodes metastases**					**0.0001**		0.162
Positive	138 (32.1%)	48.2%	48.6–47.7	70.6		13	
Negative	292 (67.9%)	84.7%	84.9–84.4	110.5		9	
**Neoadjuvant/Adjuvant treatment**							
Surgery only	57 (13.3%)	79.9%	80.5–79.2	110.4	**0.049**	0	
Neoadjuvant RT	46 (10.7%)	77.6%	78.3–76.8	111.5	**0.040**	0	
Neoadjuvant CRT	86 (20.0%)	66.4%	66.9–65.8	92.7	0.265	0	
Adjuvant CCRT	222 (34%)	69.9%	69.5–70.2	94.7	0.249	22	
Adjuvant CT	19 (4.4%)	47.5%	48.8–46.1	75.7	**0.053**	0	
**Median follow-up duration (months)**	65 (2–128)						
**Status**							
Alive	308 (71.6%)	72.4%	72.6–72.1	97.4		22	
Alive free of disease	286 (67%)						
Alive with disease	22 (5%)						
Patients who answered the questionnaires	208					0	
Deceased	122 (28.4%)						

**Table 2:** Multivariate Cox proportional hazard regression analysis of overall survival predictors.

Variables	B	SE	Wald	*p*-Value	HR	95.0% CI for OR	
						Lower	Upper
**Clinical Stage FIGO IIB**	−0.378	0.217	3.037	0.081	0.685	0.389	0.945
**Adjuvant CT**	−1.119	0.378	8.785	0.003	0.326	0.259	1.009
**Parametrial invasion**	−1.109	0.218	25.866	0.0001	0.330	0.220	0.850
**Lymph node metastasis**	−1.012	0.234	18.707	0.0001	0.363	0.234	1.001

B, regression coefficient; SE, standard error; Wald, χ^2^ value equal to B2 divided by its standard error; HR, hazard ratio; CI, confidence interval; OR, odds ratio.

**Table 3:** The QoL of the 208 patients who answered the EORTC QLQ-C30 and EORTC QLQ-CX24.

Number of Patients = 208	Items ~	Mean Score	SD *	Cronbach’s Alpha Coefficient #
**QLQ-C30**				
**Functioning scales α**				
Physical α	1–5	66.5	26.1	0.93
Role α	6, 7	67.6	27.9	0.93
Cognitive α	20, 25	66.2	27.5	0.80
Emotional α	21–24	63.1	27.4	0.94
Social α	26, 27	70.6	27.7	0.85
Global quality of life α	29, 30	64.6	25.3	0.95
**Symptom scales and/or items γ**				
Fatigue γ	10, 12, 18	37.9	26.6	0.88
Nausea and vomiting γ	14, 15	32.1	28.9	0.86
Pain γ	9, 19	32.9	28.1	0.86
Dyspnea γ	8	28.6	27.7	NA
Sleep disturbance γ	11	38.7	30.2	NA
Appetite loss γ	13	32.6	30.3	NA
Constipation γ	16	39.2	33.4	NA
Diarrhea γ	17	31.7	29.9	NA
Financial impact γ	28	35.4	31.9	NA
				
**QLQ-C24**				
Symptoms Experience γ	31–37, 39, 41–43	25.9	19.1	0.879
Body Image γ	45–47	48.4	31.3	0.946
Sexual/Vaginal Functioning γ	50–53	64.8	23.7	0.852
Lymphoedema γ	38	54.4	31.6	NA
Peripheral Neuropathy γ	40	71.7	30.4	NA
Menopausal Symptoms γ	44	70.3	32.3	NA
Sexual Worry γ	48	56.8	34.6	NA
Sexual Activity α	49	14.2	25.6	NA
Sexual Enjoyment α	54	33.4	28.2	NA

* Standard Deviation. **~** Numbers match to the item numbers in the QLQ-C30 and QLQ-CX24. # Cronbach’s alpha coefficient is considered to be a measure of validity and reliability—a value of 0.70 and above is good, 0.80 and above is better, and 0.90 and above is best [17]. **α** Scores range from 0 to 100, with a higher score indicating a higher level of functioning. **γ** Scores range from 0 to 100, with a higher score indicating a greater grade of symptoms.

## Data Availability

We provide our data for the reproducibility of this study in other centers if such is requested.
